# Iterative Adaptation of a Tuberculosis Digital Medication Adherence Technology to Meet User Needs: Qualitative Study of Patients and Health Care Providers Using Human-Centered Design Methods

**DOI:** 10.2196/19270

**Published:** 2020-12-08

**Authors:** Devika Patel, Christopher Allen Berger, Alex Kityamuwesi, Joseph Ggita, Lynn Kunihira Tinka, Patricia Turimumahoro, Joshua Feler, Lara Chehab, Amy Z Chen, Nakull Gupta, Stavia Turyahabwe, Achilles Katamba, Adithya Cattamanchi, Amanda Sammann

**Affiliations:** 1 Department of Surgery University of California, San Francisco San Francisco, CA United States; 2 Division of Pulmonary and Critical Care Medicine and Center for Tuberculosis University of California, San Francisco San Francisco, CA United States; 3 Uganda Tuberculosis Implementation Research Consortium Kampala Uganda; 4 School of Medicine Yale University New Haven, CA United States; 5 Everwell Health Solutions Bangalore India; 6 Uganda National Tuberculosis and Leprosy Programme Kampala Uganda; 7 Department of Medicine Makerere University College of Health Sciences Kampala Uganda

**Keywords:** human-centered design, tuberculosis, digital adherence technology, digital health, adherence, medication, treatment, outcome, lung, respiratory, infectious disease

## Abstract

**Background:**

Digital adherence technologies have been widely promoted as a means to improve tuberculosis medication adherence. However, uptake of these technologies has been suboptimal by both patients and health workers. Not surprisingly, studies have not demonstrated significant improvement in treatment outcomes.

**Objective:**

This study aimed to optimize a well-known digital adherence technology, 99DOTS, for end user needs in Uganda. We describe the findings of the ideation phase of the human-centered design methodology to adapt 99DOTS according to a set of design principles identified in the previous inspiration phase.

**Methods:**

99DOTS is a low-cost digital adherence technology wherein tuberculosis medication blister packs are encased within an envelope that reveals toll-free numbers that patients can call to report dosing. We identified 2 key areas for design and testing: (1) the envelope, including the form factor, content, and depiction of the order of pill taking; and (2) the patient call-in experience. We conducted 5 brainstorming sessions with all relevant stakeholders to generate a suite of potential prototype concepts. Senior investigators identified concepts to further develop based on feasibility and consistency with the predetermined design principles. Prototypes were revised with feedback from the entire team. The envelope and call-in experience prototypes were tested and iteratively revised through focus groups with health workers (n=52) and interviews with patients (n=7). We collected and analyzed qualitative feedback to inform each subsequent iteration.

**Results:**

The 5 brainstorming sessions produced 127 unique ideas that we clustered into 6 themes: rewards, customization, education, logistics, wording and imagery, and treatment countdown. We developed 16 envelope prototypes, 12 icons, and 28 audio messages for prototype testing. In the final design, we altered the pill packaging envelope by adding a front flap to conceal the pills and reduce potential stigma associated with tuberculosis. The flap was adorned with either a blank calendar or map of Uganda. The inside cover contained a personalized message from a local health worker including contact information, pictorial pill-taking instructions, and a choice of stickers to tailor education to the patient and phase of treatment. Pill-taking order was indicated with colors, chevron arrows, and small mobile phone icons. Last, the call-in experience when patients report dosing was changed to a rotating series of audio messages centered on the themes of prevention, encouragement, and reassurance that tuberculosis is curable.

**Conclusions:**

We demonstrated the use of human-centered design as a promising tool to drive the adaptation of digital adherence technologies to better address the needs and motivations of end users. The next phase of research, known as the implementation phase in the human-centered design methodology, will investigate whether the adapted 99DOTS platform results in higher levels of engagement from patients and health workers, and ultimately improves tuberculosis treatment outcomes.

## Introduction

### Background

Tuberculosis (TB) is now the leading infectious killer globally, with an estimated 10 million people falling sick annually and nearly 1.5 million dying of the disease [1]. Patient adherence to and completion of TB treatment is a key barrier to reducing mortality and amplification of drug resistance [2]. Recent data suggested that adherence rates of less than 90%, or missing more than 3 doses in a month, can decrease treatment success and cure rates [3]. Directly observed therapy (DOT), wherein a health worker observes a patient taking each daily dose of TB medicines, has been a core component of the World Health Organization (WHO)-recommended strategy for supervising TB treatment [4]. However, DOT is associated with numerous challenges, including questionable effectiveness in improving treatment outcomes [5,6], as well as time and cost burdens for patients and health workers [7,8].

Digital adherence technologies (DATs) have emerged as an alternative to facilitate and promote TB treatment adherence and completion [9-11]. The range and breadth of DATs have expanded rapidly as companies continue to develop unique technological platforms and devices, including (1) short message service (SMS) text message reminders; (2) “dose-in-hand” event monitors, including electronic pill boxes [12,13] and sleeves that fit over medication blister packs [14]; (3) video-observed treatment to remotely record pill ingestion using smartphones; and (4) wireless-observed treatment, in which silicon sensors embedded in TB pills document ingestion [11,15]. Each of these products connects with virtual platforms that allow providers to view real-time adherence data for their patients.

Despite the use of DATs in TB programs [16], supporting data are limited and show minimal to no improvement in treatment success and mortality [9,17]. Additionally, several large-scale implementation studies have shown suboptimal patient engagement with various DATs [18-20]. In response, the WHO and other large funding agencies have called for country-level assessments and stakeholder mapping to support rollout of and user engagement with DATs [15,21].

This study was the second phase in the adaptation of the 99DOTS envelope using the human-centered design (HCD) methodology. HCD is a well-established problem-solving and innovation methodology with the potential to improve adoption and implementation challenges that have impeded the impact of DATs. HCD has been employed in a variety of industries over the past 40 years, notably in business and technology [22-25]. This approach uses qualitative research methods to understand the values and motivations of users, and to systematically incorporate user input to guide the design or adaptation of products and interventions [24]. The primary focus on the user throughout the entire process—problem framing, solution development, implementation, and evaluation—may yield more efficacious and sustainable results [26].

99DOTS is a novel sleeve-based DAT wherein TB medication blister packs are housed within an envelope that reveals hidden, toll-free numbers when pills are pushed out. Patients call these toll-free numbers daily to confirm medication dosing. Clinic staff can access real-time dosing information from both a web and mobile phone app to guide patient follow-up. SMS text messaging reminders, both to patients for dosing and to providers to follow up on patients who have missed doses, are also a core feature of the platform [13,14].

### Objective

The first phase of this study used HCD’s approach to human factors research to identify key insights and design opportunities for modifying the design of the original 99DOTS product [27]. The purpose of this second phase of the study was to address these design opportunities through modification of key features of the 99DOTS platform in order to improve TB medication adherence in Uganda.

## Methods

### Context and Setting

This was the second phase of a 3-phase study using HCD methodology to adapt the 99DOTS platform [13]. In HCD, these phases are termed inspiration, ideation, and implementation. Previously, in the inspiration phase, we conducted 67 semistructured interviews with TB patients, family members, health workers, and community leaders at 8 rural and periurban TB treatment centers in Uganda. Key themes and quotes were elicited from interviews and translated into actionable insights and design opportunities (Table 1) [27]. Here, we report on the ideation phase, which focused on the development and testing of solutions in order to address the insights and design opportunities identified in the inspiration phase*.* In reporting qualitative aspects of the study, we followed the Consolidated Criteria for Reporting Qualitative Research (COREQ-2) checklist (Multimedia Appendix 1) [28]. The study was approved by the University of California San Francisco, Committee on Human Research, San Francisco, CA, USA (protocol number 221678, June 11, 2018) and the Makerere University School of Public Health Research Ethics Committee, Kampala, Uganda (protocol number HS2436, July 19, 2018).

**Table 1:** Insights and opportunities from the inspiration phase.

Insight	Opportunity
1. Social stigma may be feared as much as the disease.	Make tuberculosis treatment discreet by avoiding nonconspicuous labels and visuals.
2. Packaging is used as an ad hoc and accidental reminder system.	Guide sequential pill-taking behavior and accurately track treatment milestones.
3. Health is defined by a return to normal strength and capability, which occurs before treatment completion.	Reinforce the importance of treatment completion to sustain health at every patient point of contact.
4. Feeling a personal connection with health care workers is as important as receiving medical care.	Leverage the relationship between patients and community health workers to personalize the treatment experience.
5. Local messages feel authentic; endorsements by famous figureheads are viewed with suspicion.	Incorporate authentic and personal adherence messages from the local community in the tuberculosis treatment program.
6. Patients are motivated by service to their community, but do not have enough knowledge education to be maximally effective.	Empower individuals to be community stewards of infection control practices through language and literacy appropriate education.
7. Words and visuals can have multiple interpretations and an unintended negative impact on adherence behavior.	Communicate clearly and accurately with simple graphics and culturally and linguistically appropriate text.

### 99DOTS System and Redesign Opportunities

**Figure 1:**
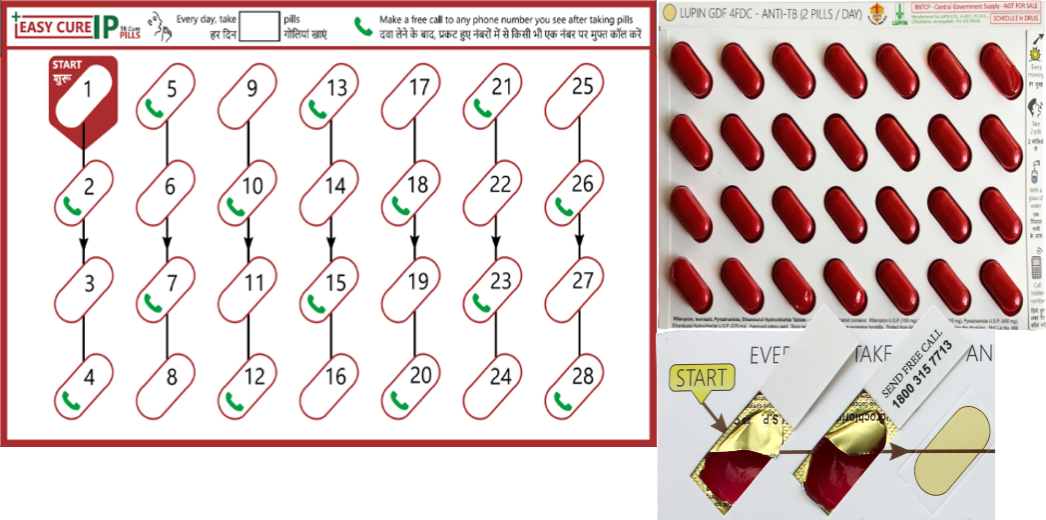
The original 99DOTS product consists of an envelope that fits around the tuberculosis medication blister pack distributed to patients. When patients remove pills from the blister pack each day, a hidden toll-free phone number is revealed. Patients call the number to record medication ingestion, and all calls are logged as a taken dose on the 99DOTS platform. Providers log in to an online dashboard to access patients’ adherence history via a desktop or mobile phone app. Text messages are sent to patients daily to remind them to take their medications and to providers to alert them about patients who have missed consecutive doses.

This study focused on the redesign of 2 components of the original 99DOTS platform: (1) the pill packaging envelope that reveals hidden, toll-free numbers called by patients to report dosing and (2) the patient experience when calling toll-free numbers (Figure 1)*.* In the original platform, pills are visible on one side of the pill packaging envelope and, as pills are pushed out of the blister pack, a toll-free number is revealed on the opposite side. Upon calling the toll-free number patients hear a beeping sound and the call is logged by the 99DOTS server to indicate a taken dose. The process is repeated on each day the patient is scheduled to take TB medications. A daily dosing history is thus created that providers can access by logging in to an online dashboard via a desktop or mobile phone app and can be used to send SMS text message alerts to providers to notify them of missed consecutive doses. Adaptable features associated with the original pill packaging envelope included its form factor (physical packaging design and external images), content (instructional education and messaging), and visuals (wayfinding instructions for pill taking and revealed phone numbers). The adaptable feature associated with the patient call-in experience was what patients hear when making calls to self-report dosing.

### Prototype Development

From the 5 brainstorming sessions informed by the interviews conducted in the previous phase and structured around the design opportunities identified in the inspiration phase, we built a diverse list of unique concepts that we could develop into prototypes. Each session started with a “How might we...?” question that was written to maximize brainstorming output without suggesting a solution [29]. Brainstorming sessions included 3 designers and at least 2 other members of the research team. Each participant was given 3 minutes to generate and capture ideas on sticky notes. The ideas were shared and displayed on foam boards. Participants collaboratively generated additional ideas and discussed whether any potential ideas had been missed. When all 5 brainstorming sessions were complete, we reviewed each idea and clustered redundant or related ideas to represent discrete concepts that we could develop into a prototype. We then organized the concepts into more general themes. Written notes and photographs of the thematic displays captured the output of each brainstorming session.

The general themes and associated concepts were presented to senior investigators (AS, AC, and AKa), who selected the concepts that had the most promise to be developed into prototypes based on the following criteria: (1) whether the concept was clearly linked to a design opportunity identified in the previous phase of research; (2) whether the concept could be applied to one of the adaptable 99DOTS features, and (3) whether the concept was feasible based on the constraints of the 99DOTS platform and available resources. We selected a minimum of 4 concepts addressing at least 2 different design opportunities for each feature of the pill packaging and for the patient call-in experience. Pill packaging prototypes were developed by visual designers using Adobe Creative Cloud (Adobe Inc), and audio messages for the patient call-in experience were drafted in Microsoft Word (Microsoft Corporation). These prototypes were then iteratively revised by team members in Uganda and the United States until consensus was reached. Each prototype selected for field testing was translated from English into Luganda, the primary language spoken at the study research sites.

### Prototype Testing

#### Participant Recruitment

The prototypes were tested in September 2018 by 1 design researcher and 5 local research staff. We recruited health workers and patients (users) for testing from 18 Uganda National Tuberculosis and Leprosy Program TB treatment units participating in a stepped-wedge randomized trial of 99DOTS. To ensure a diverse representation of treatment units, we recruited at least one TB care provider from each of the 18 health centers. All health workers involved in providing TB treatment who attended the public randomization ceremony for the parent trial were invited to participate in a focus group. Prototypes were tested in 5 focus groups with 52 health workers, with each focus group consisting of 9 to 13 participants. During the health facility site visits, we also conducted semistructured interviews with all patients who presented for a TB treatment follow-up visit (n=7). Focus groups were conducted in English, and patient interviews were conducted in English or Luganda, depending on patient preference.

#### Pill Packaging Envelope

We used a standardized questionnaire (Multimedia Appendix 2) in focus groups and interviews to compare a series of up to 6 prototypes for each feature of the pill packaging envelope. Positive and negative feedback were obtained for each prototype, and users were asked to propose changes to improve each prototype. Feedback received during both focus groups and interviews was recorded in written notes by Ugandan research staff, then reviewed independently by 2 US researchers and clustered into themes.

#### Patient Call-In Experience

We first tested audio message prototypes in focus groups with health workers during the randomization ceremony. Audio messages were presented by thematic category and read aloud in English. Feedback was solicited after each message, which included real-time edits to the language, suggestions for improvement, and removal of messages that did not resonate with users. The revised messages were then clustered into themes and further evaluated through interviews with patients. Patients were asked what audio messages they would like to hear from their health care workers and then given examples of the different messaging themes generated from the health worker focus groups.

### Prototype Iteration

We analyzed qualitative feedback collected during prototype testing to inform the next set of changes to each component. We analyzed the resulting interview transcripts using an inductive approach to thematic analysis [30]. All transcripts were read independently by 2 to 3 members of the research team to identify key themes across the interviews. The reviewers then met to review all thematic categories and ensure that accurate descriptions and supporting quotes depicting positive and negative user feedback were assigned to each theme. Following each iteration, the prototype was refined based on the list of themes that emerged from user feedback, alignment with a particular design opportunity, and feasibility of implementation in the 99DOTS system. We used the final list of themes from each testing session to refine each prototype. The final designs for the pill packaging components and the patient call-in experience were revised over multiple cycles of iteration and feedback from the entire research team, including Uganda- and US-based personnel, until consensus was reached.

## Results

### Brainstorming

**Figure 2:**
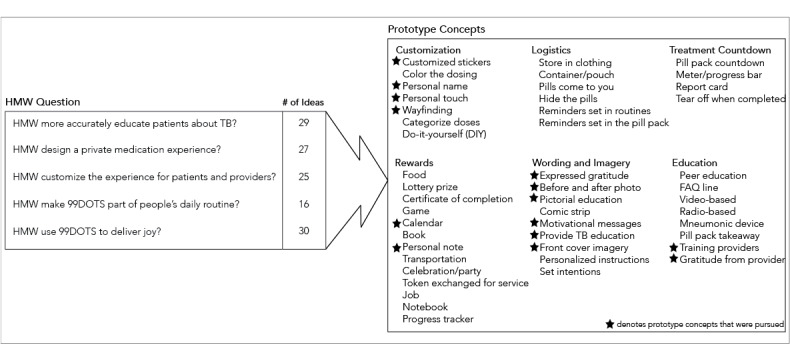
A total of 5 brainstorming sessions centered on one “How might we?” (HMW) question (left). Participants were each allowed 3 minutes to put 1 unique idea on a sticky note, which was later visibly displayed to the group and summated under each HMW question. After all brainstorm sessions were complete, ideas were clustered into 6 themes: customization, rewards, logistics, wording and imagery, treatment countdown, and education. Those ideas that addressed the design opportunities and were feasible to implement moved forward for testing (designated by a star). FAQ: frequently asked questions; TB: tuberculosis.

Brainstorming sessions addressed the following “How might we...?” questions: (1) “How might we more accurately educate patients about TB?”; (2) “How might we design a private medication experience?”; (3) “How might we customize the experience for patients and providers?”; (4) “How might we make 99DOTS part of people’s daily routine?”; and (5) “How might we use 99DOTS to deliver joy?” Overall, the brainstorms generated 127 unique ideas. The first session, on education, generated 29 ideas; the second session, on privacy, generated 27 ideas; the third session, on customization, generated 25 ideas; the fourth session, on daily routine, generated 16 ideas; and the fifth session, on delivering joy, generated 30 ideas. We clustered the ideas into 47 unique concepts grouped into 6 major themes: rewards (14 prototype concepts), customization (7 prototype concepts), education (8 prototype concepts), logistics (5 prototype concepts), wording and imagery (9 prototype concepts), and treatment countdown (4 prototype concepts) (Figure 2). These themes were linked back to the design opportunities identified in the previous phase of research (Table 1) to identify which themes had concordance with the design opportunities. This, in turn, informed the prototypes chosen for development and testing.

### Prototype Development

**Figure 3:**
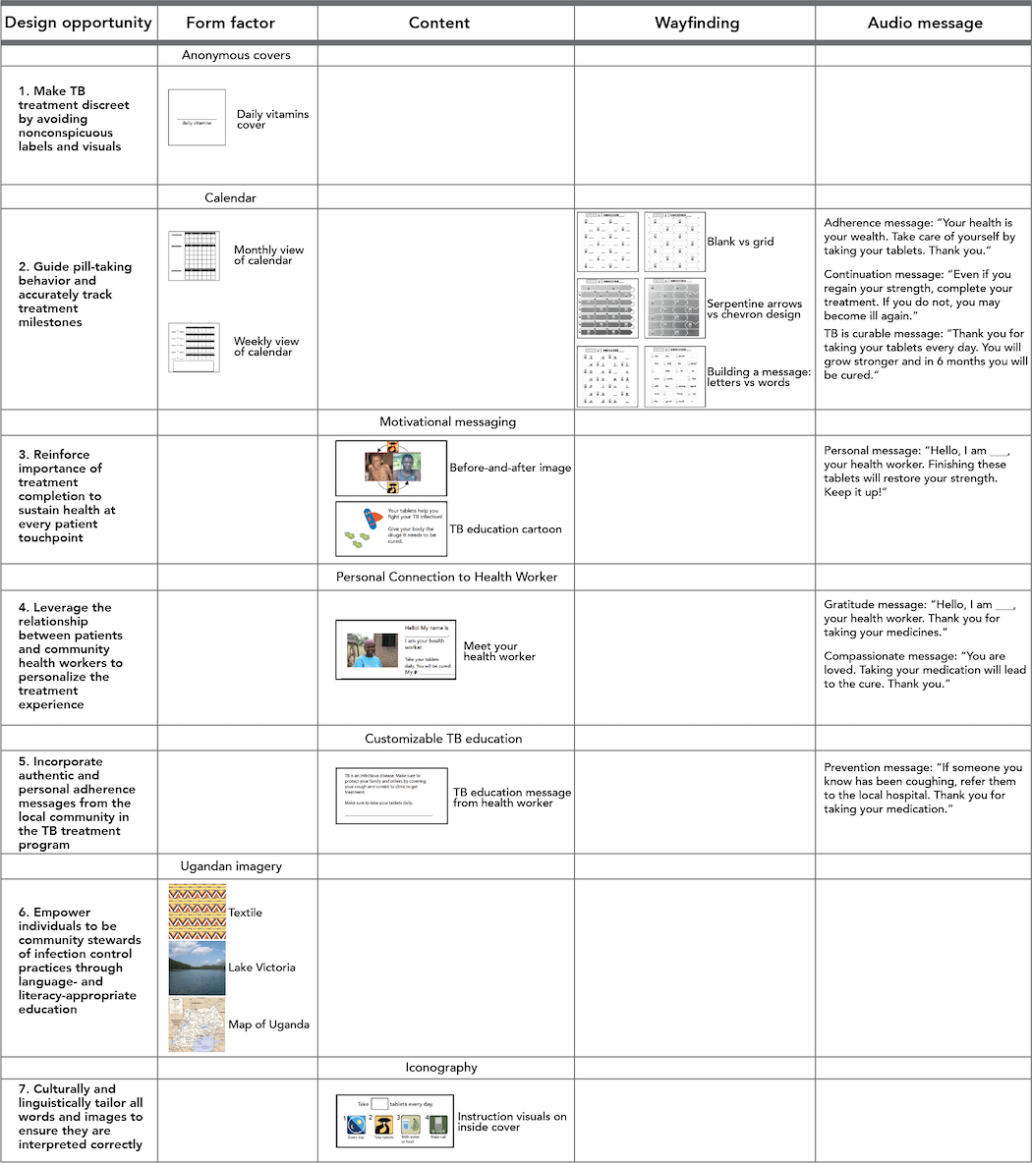
For each design opportunity, we prototyped different form factors, content, wayfinding, and motivational messaging. TB: tuberculosis.

We developed 16 packaging prototypes, 12 icons, and 28 messages for testing. For each prototype, Figure 3 indicates the design opportunity addressed and the adapted 99DOTS feature.

#### Pill Packaging Envelope Form Factor

Given the importance of discretion (design opportunity 1; Figure 3), we determined that the pills had to be covered. In collaboration with our 99DOTS production partners, we decided that the most feasible design was an additional front flap to cover the pill pack. We subsequently developed 6 discrete prototypes for the front flap imagery: (1) 1 anonymous cover (a daily vitamins label) that addressed discretion (design opportunity 1); (2) 2 calendar covers (weekly and monthly) to track treatment milestones (design opportunity 2); and (3) 3 covers with Ugandan imagery (a map of Uganda, Lake Victoria, and a Ugandan textile design) that addressed national and community pride (design opportunity 6).

#### Pill Packaging Envelope Content

We developed 4 prototypes for the inside cover. These were (1) 2 educational messages about treatment completion: a before-and-after treatment photo of a patient with TB, and a cartoon reinforcing the importance of TB treatment completion (design opportunity 3) and (2) 2 personal messages: 1 introductory message from a health worker to reinforce their relationship (design opportunity 4) and 1 tailored, personal message from the health worker (design opportunity 5). We also developed 12 unique icons to illustrate the instructions for taking the medication: 2 icons for illustrating daily dosing, 6 icons to illustrate taking pills by mouth, and 4 icons to represent a toll-free phone call.

#### Pill Packaging Envelope Wayfinding

To assist patient understanding of the order in which to take pills, we developed 4 visual prototypes: a serpentine pattern, a chevron pattern, a customizable pattern, and an unannotated blister pack (design opportunity 2).

#### Patient Call-In Experience

Given the importance of encouraging adherence at every contact point with a patient (design opportunity 3), we determined that the call-in experience would benefit from adaptation from the original system. Originally, the call concluded with a short “thank you” before its automatic termination. We adapted this feature to include a changing daily audio message. Messages were developed to represent 7 themes: gratitude, adherence, reminders, prevention, encouragement, reassurance that TB is curable, and compassion from health care workers (design opportunities 3, 4, 5, and 6).

### Prototype Testing

We tested prototypes with 59 users overall: 5 focus groups comprising 52 health workers representing all 18 health centers enrolled in the stepped-wedge trial, and 7 patients at 2 of the 18 health centers.

#### Pill Packaging Envelope Form Factor

When we tested the anonymous cover option, users did not view the daily vitamins label favorably. Although mislabeling the cover as daily vitamins was perceived to avoid stigma, health workers and patients were concerned it would lead to unintended use by children or other members of the family. One patient noted: “Those who are not sick would want to take the vitamins.”

Testing of the 2 calendar cover options (weekly vs monthly calendar) revealed that patients universally liked the idea of the calendar to track their daily dosing and treatment. One patient noted: “The calendar can show me the days I have taken the medicine.” However, there was no clear preference for the weekly or monthly option. Health workers expressed mixed sentiments. Some liked that it would help patients track their medications, whereas others were concerned that it would duplicate work they are already doing: “It would be extra work for patient and health care worker.”

Of the 3 Ugandan imagery prototypes tested, most patients (5 of 7) preferred the map of Uganda over the images of Lake Victoria and a Ugandan textile. There was no consensus among health workers. However, patients and health workers believed the map was useful because it indicated that TB treatment is a national effort, and patients stated that they would use the map for directions when traveling: “It is our country, if I am going anywhere I can refer to the map.”

#### Pill Packaging Envelope Content

Among the educational messages tested, the before-and-after treatment photo of a patient with TB was preferred to the TB treatment cartoon. Health workers believed the before-and-after photo was a powerful reminder of the consequence of nonadherence but were still concerned that it might propagate stigma: “It is a beautiful message but can lead to stigma if someone else sees it.” In the before-and-after photo of a patient with TB, we used circular arrows to show the link between the before image and the after image. Health workers did not like the circular arrows because they suggested a cycle and were concerned that the image represented an extreme case of TB that might not be relevant to the majority of patients: “The picture shows the worst stage you can die, not all patients get wasted like that.” However, all patients believed the images were motivating: “It encourages me to take my medicine; if I don’t take, I will look like this.” Conversely, the TB treatment cartoon was misunderstood by every patient.

There was enthusiasm among both health workers and patients for multiple personal messages. Connecting patients directly to their health worker through the health worker introduction was favored by all patients, especially the inclusion of the health worker’s phone number: “I can easily call or consult my health worker.” The tailored message was universally liked because it addressed patient concerns such as protecting their family and educating others: “If another person has the condition, I can show it.” Patients and health care workers liked the space for custom messages and stated that they wanted their health worker to “add that TB is curable to give hope.” Two patients and all providers requested that the education have more pictures and less text to support individuals with language and literacy challenges.

We tested the 12 unique icons against each other in each category. There was no consensus on the daily dosing icon. Patients liked the sun image because it implied that medications were to be taken during the day. Health care workers preferred the image with a sun and moon because it allowed customization of the dosing time but were concerned that it might be misinterpreted as twice daily dosing. There was universal consensus on the take-pills-by-mouth image, where the majority preferred the image with the side profile and hand holding the pill. For the phone call icon, patients preferred the image with the phone and “0 UGX” (ie, 0 Ugandan schillings) at the bottom because it was most clear. Providers either had no preference or agreed with the patient choice. The biggest concern remained the need to reinforce that this was a toll-free call.

#### Pill Packaging Wayfinding

Patients and health workers preferred the graphics that clearly defined the direction of pill taking, but there was no consensus on whether this should include the chevron or serpentine pattern. Instead of taking the pills in a left-to-right direction across rows, they preferred to take the pills from the top to the bottom of the pill pack. Patients and health workers preferred color to black-and-white prototypes.

#### Patient Call-In Experience

We tested 7 themes of audio messaging. The themes that were universally preferred by health workers and patients were reassurance that TB is curable; prevention, especially messages about how to protect others; and encouragement. Patients also preferred compassionate messages from health workers: “Ask if I am ok, have I treated my body well?” Providers additionally preferred messages about adherence and appointment reminders.

### Iteration to Final Prototype

**Figure 4:**
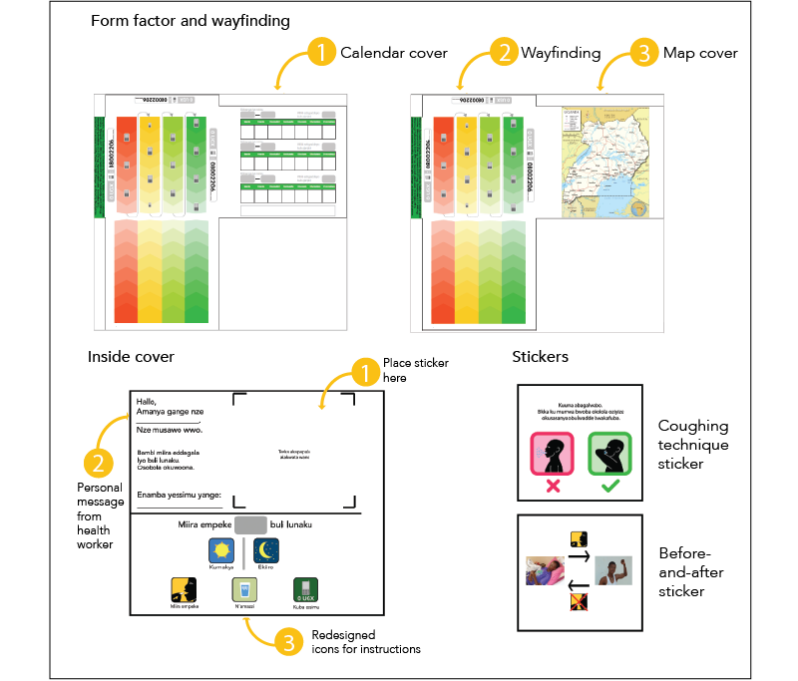
Final prototypes of the form factor, content, and wayfinding for the intensive phase of tuberculosis (TB) treatment (first 2 months). A front flap was added to the envelope to conceal the medications with an image of either a calendar (1) or a map of Uganda (3). The wayfinding (2) used color and a chevron pattern with mobile phone icons to indicate dosing days and pill-taking order. The inside covers were customized to include a personal message and contact information for local health workers (2), pictorial pill-taking instructions (3), and several sticker designs to be chosen by the patient (1). Intensive-phase stickers focused on coughing technique when a person may still be infectious and the importance of medication adherence to return to health. Translation of Luganda: personal messages from health worker: “Hello! My name is _____. I am your health worker. Please take your tablets daily. You can be cured! My telephone number: _____”; coughing technique sticker: “Protect your loved ones. Cover your mouth when you cough to prevent the spread of TB.”; blank sticker space: “Place sticker here”; icons: “Take ____ tablets every day”, “Morning”, “Night”, “Take tablets”, “With water”, “Make call”.

**Figure 5:**
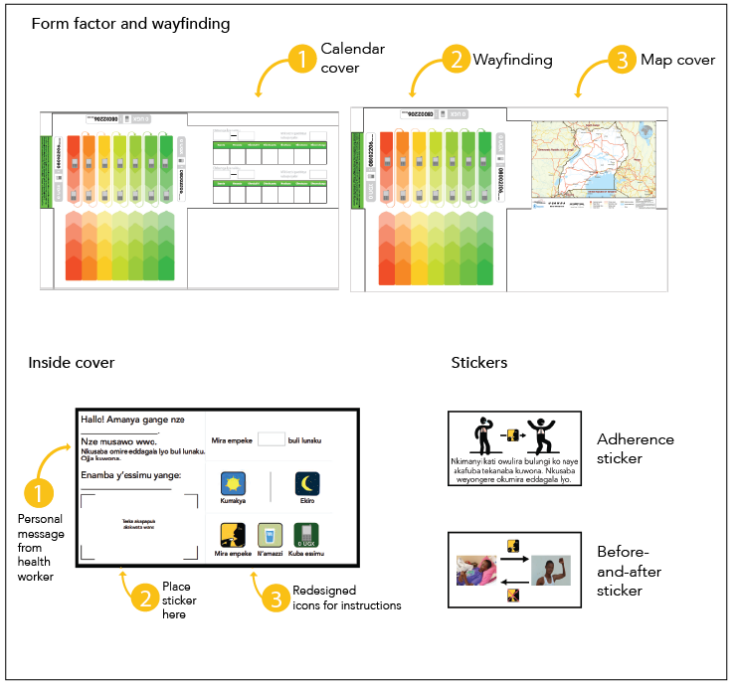
Final prototypes of the form factor, content, and wayfinding for the continuation phase of tuberculosis (TB) treatment (final 4 months). A front flap was added with an image of either a calendar (1) or a map of Uganda (3). The wayfinding (2) used color and a chevron pattern with mobile phone icons to indicate dosing days and pill-taking order. The inside covers were customized to include a personal message and contact information for local health workers (1), pictorial pill-taking instructions (3), and several sticker designs to be chosen by the patient (2). Both continuation phase stickers focused on the importance of medication adherence, even when a person is feeling better, until the end of treatment. Translation of Luganda: personal messages from health worker: “Hello! My name is ______. I am your health worker. Please take your tablets daily. You can be cured! My telephone number: ______”; adherence sticker: “I know you feel better now, but TB hasn’t yet been cured. Please continue taking your medicines.”; blank sticker space: “Place sticker here”; icons: “Take _____ tablets every day”, “Morning”, “Night”, “Take tablets”, “With water”, “Make call”.

We adapted the final prototype described here for the 2 phases of TB treatment (initiation and continuation), which have different pills, doses, and pill packaging shapes (Figures 4 and 5)*.* The form factor included a cover flap with 2 different image options (a map of Uganda or a weekly calendar). This design maintained discretion (design opportunity 1) by providing an unlabeled cover. The covers had more than 1 image to avoid the risk that, with time, a single image would become associated with TB.

The final pill pack content included a number of unique features:

- A compassionate message from the health worker with space to write the health worker’s name and phone number in order to support this important relationship (design opportunity 4) and to personalize the pill package experience (design opportunity 5).
- Instructional visual icons, which went through multiple iterations of changes to ensure appropriate interpretation (design opportunity 7). Notable changes included separate icons of a sun and moon to allow patients to select their dosing time, and an icon to indicate that medications should be taken with water so as not to risk nonadherence for people experiencing food insecurity.
- A customizable section on the inside flap with an option to choose 1 of 3 educational or motivational stickers: a before-and-after picture to reinforce treatment adherence (design opportunity 3), which we adapted from feedback to include a less cachectic person and bidirectional rather than circular arrows (design opportunity 7); a cough technique illustration tailored for patients in the initiation phase to support infection control (design opportunity 6); and an illustration tailored for patients in the continuation phase showing that treatment completion leads to cure (design opportunity 3) (Figures 4 and 5).

All of these images prioritized visual graphics over text per our user feedback and design opportunity 7.

The wayfinding graphics were multicolored with a graduated chevron pattern and serpentine directional arrows to guide pill taking (design opportunity 2). We adjusted this pattern to honor the cultural norm by changing the direction of pill taking to flow from top to bottom in columns and included colors that had been tested and approved by patients (design opportunity 7).

A total of 20 audio messages were recorded by local health workers (Textbox 1)*.* We recorded 10 general messages, each beginning with a personal introduction: “Hello, I am your health worker...” (design opportunities 4 and 5). The themes of the general messages included gratitude, that TB is curable, and the importance of adherence. We tailored 5 messages to patients in the initiation phase with the themes of prevention and that TB is curable, and 5 messages to the continuation phase with themes of gratitude and adherence.

**Final audio messages.**

**GENERAL (10 messages played through entire treatment):**

**Gratitude**

- Hello, I am ______ your health worker. Thank you for taking you medicines.

**Adherence**

- Hello, I am ______ your health worker. If you finish taking all medicines, you will be cured.
- Hello, I am ______ your health worker. It is essential that you take your medicines. It will lead you to the cure.
- Hello, I am your health worker. Continue to take your tablets each and every day!

**Encouragement**

- Hello, I am your health worker. Do not lose hope!
- Hello, I am ______ your health worker. Finishing these tablets will restore your strength. Keep it up!

**Compassion from health worker**

- Hello, I am your health worker. We believe in you.

**Prevention**

- Hello, I am your health worker. Cover your cough and take your medicines to protect your family and friends.
- Hello, I am your health worker. Please contact me if you feel unwell.

**Sputum checkup (time specific)**

- This is your reminder to go to clinic and get your sputum checkup. This will help us know whether you are getting better or not.

**INITIATION MESSAGES (5 messages only played in initiation phase):**

**Reassurance that tuberculosis (TB) is curable**

- TB is curable if you take your medicine following the health workers’ instructions.
- Thank you for taking your tablets every day. You will grow stronger and in 6 months you can be cured.
- The infection is curable. Remember, you will not have it for life.

**Prevention**

- Protect your family and friends. Please take your tablets daily.
- Please bring coughing relatives with you for checkup.

**CONTINUATION (5 messages only played in continuation phase)**

**Adherence**

- Thank you for taking your medication. By taking your medication every day, you can be cured at the end of 6 months.
- If you don’t take all of your pills, you can get sick again. Thank you.
- You may not feel sick, but TB is still in your body. Please take your tablet for the remaining months to be cured.
- TB can come back if you do not complete your treatment. Take your tablets every day.

**Prevention**

- Prevention begins with you. Complete your treatment and ensure your health.

## Discussion

### Principal Findings

In this study representing the ideation phase of HCD research, we adapted existing features of 99DOTS—a low-cost DAT—to better meet user needs and impact TB medication adherence in Uganda. We leveraged the insights and design opportunities identified from the previous inspiration phase to guide tangible, user-centered design alterations to the 99DOTS platform [27,31]. We first identified key components of the 99DOTS platform for customization: the envelope and patient call-in experience. Through a series of brainstorming sessions, we developed a wide range of ideas that we then narrowed based on feasibility and grounding in the outlined design opportunities. We then field tested the resulting prototypes through iterative cycles of feedback and design to create the final prototype that we used in an ongoing stepped-wedge trial of the 99DOTS technology in Uganda.

Current data indicate that the potential impact of DATs has been hindered by a failure to meet the needs and desires of patients and health workers [18,32,33]. As widespread deployment of DATs continues in several high-burden TB countries [14,19], multinational agencies have called for more detailed stakeholder engagement in an effort to adapt these technologies [15,21,34]. The disconnect between currently available technologies and the needs and desires of users has been notable in multiple settings [18,20,33,35-38]. Specifically, evaluations of the 99DOTS platform have highlighted reduced patient contact [18,19], a lack of personalized engagement between providers and patients [20,39], limited patient literacy [33], and ongoing stigma resulting in health care avoidance as key barriers to the successful rollout of 99DOTS [20,38]. In addition, the inspiration phase of our research further highlighted the use of packaging as a reminder system (insight 2), the importance of defining health per local constructs (insight 3), and the need to educate patients about their disease (insight 6). To address these barriers to the adoption and implementation of 99DOTS, our adapted 99DOTS platform includes visuals to guide pill-taking behavior, discreet images depicting messaging about health as a return of strength, and culturally and linguistically tailored educational and motivational messages from health workers.

The WHO’s *Handbook for the Use of Digital Technologies to Support Tuberculosis Medication Adherence* recommends that, when implementing digital health solutions, it is necessary for implementors to thoroughly assess the context of the health care system and position the patients at the center, with their needs and challenges incorporated into the implementation and decision-making process [11]. HCD, when done correctly, facilitates this process by providing a structured, user-centered approach to iteratively develop solutions that target barriers and facilitators identified in formative research. Determining how best to address barriers and facilitators is a key challenge for implementation science. Our previous experience [40], as well as that of others, shows that implementation failures are common, even when interventions are informed by implementation theories or frameworks [41-43]. To overcome this challenge, HCD allows for a broad range of solutions to be considered and either rejected or refined through user testing prior to large-scale implementation studies. Although HCD is relatively new in global health research, there are now several examples of HCD-informed approaches in addressing implementation problems. In Kenya, Catalani et al used HCD to create a TB clinical decision support system to improve uptake of TB prevention therapy among people living with HIV [26], and a group in Colombia recently demonstrated the use of HCD to develop 4 novel prototypes to improve cervical cancer screening [44]. These examples, in addition to our work, add to the growing literature on the potential benefits of using the HCD approach in addressing implementation barriers.

### Limitations

Our study has several important limitations. The outputs of brainstorming sessions in part reflect the participants included. While we did not include patients and health workers for logistical reasons, the sessions included a diverse group of designers and researchers, including Ugandan social scientists and public health researchers. The relatively small number of patients available to be interviewed for prototype testing is a limitation, though patient perspectives obtained during the prior phase of research informed the initial prototypes. We plan for additional patient interviews to obtain further feedback during the subsequent implementation phase of the project.

### Conclusions

We adapted a DAT to the expressed needs and desires of health workers and TB patients using HCD methodology. The results of our ongoing trial will confirm whether the adaptations made to strengthen connections between 99DOTS and its intended users result in improved TB medication adherence and treatment outcomes.

## Appendix

Multimedia Appendix 1 COREQ-2 checklist.

Multimedia Appendix 2 Focus-group standardized questionnaire.

## Abbreviations

DAT: digital adherence technology
DOT: directly observed therapy
HCD: human-centered design
SMS: short message service
TB: tuberculosis
WHO: World Health Organization
