# Supplementary material for: Iterative Adaptation of a Tuberculosis Digital Medication Adherence Technology to Meet User Needs: Qualitative Study of Patients and Health Care Providers Using Human-Centered Design Methods
Source: JMIR Form Res. 2020 Dec 8;4(12):e19270. doi: 10.2196/19270 (PMC7755538; doi:10.2196/19270)
Supplement: Multimedia Appendix 1 [file formative_v4i12e19270_app1.pdf]

Consolidated criteria for reporting qualitative studies (COREQ): 32-item checklist

No	Item	Guide questions/description	Study Team Responses
**Domain 1: Research team and reflexivity**			
**Personal Characteristics**			
**1.**	**Interviewer/facilitator**	**Which author/s conducted the interview or focus group?**	**Patient Interview:**<br>**1) DP 2) AKi**<br>**Provider Interview:**<br>**1) DP 2) Aki 3) PT 4) JG**
**2.**	**Credentials**	**What were the researcher's credentials?** ***E.g. PhD, MD***	**DP – Design Degree and MS in Community Health and Prevention Research**<br>**AKi – Medical Officer**<br>**PT – Medical Officer**<br>**JG – Masters in Qualitative Research**
**3.**	**Occupation**	**What was their occupation at the time of the study?**	**DP – design researcher**<br>**Aki – project manager**<br>**PT – project manager**<br>**JG – research assistant**
**4.**	**Gender**	**Was the researcher male or female?**	**DP – Female**<br>**Aki – Male**<br>**PT – Female**<br>**JG - Male**
**5.**	**Experience and training**	**What experience or training did the researcher have?**	**DP – 5 years of qualitative design research**<br>**Aki – 2 years of qualitative design research**<br>**PT – 3-5 years of qualitative research**<br>**JG – masters in qualitative research**
**Relationship with participants**			
**6.**	**Relationship established**	**Was a relationship established prior to study commencement?**	**Yes - all 18 health centers and their staff has been enrolled in a stepped-wedge trial for evaluation of adherence technology. The interviews were done at the randomization ceremony for the parent study.**

**7.**	**Participant knowledge of the interviewer**	**What did the participants know about the researcher? e*.g. personal goals, reasons for doing the research***	**All providers had been at a day long training to learn the objectives of the study. Patients were read a script and consent form which detailed the overall objective of this sub study.**
**8.**	**Interviewer characteristics**	**What characteristics were reported about the interviewer/facilitator? e.g. *Bias, assumptions, reasons and interests in the research topic***	**Each interviewer introduced themselves to the participants including their name, occupation, and country of origin.**
**Domain 2: study design**			
**Theoretical framework**			
**9.**	**Methodological orientation and Theory**	**What methodological orientation was stated to underpin the study? *e.g. grounded theory, discourse analysis, ethnography, phenomenology, content analysis***	**Human-centered design utilized grounded theory and an inductive approach to content analysis.**
**Participant selection**			
**10.**	**Sampling**	**How were participants selected? *e.g. purposive, convenience, consecutive, snowball***	**All providers at the training were invited to participate (no sampling). A convenience sample of patients from 2 nearby health centers were included with an effort to purposely sample a range based on age, gender, HIV status, and treatment stage.**
**11.**	**Method of approach**	**How were participants approached? e*.g. face-to-face, telephone, mail, email***	**Clinic staff, who have a personal relationship with patients, approached participants face-to-face at regular refill visits to ask if they would like to participate.**
**12.**	**Sample size**	**How many participants were in the study?**	**52 providers and 7 patients were interviewed.**
**13.**	**Non-participation**	**How many people refused to participate or dropped out? Reasons?**	**None.**
**Setting**			

**14.**	**Setting of data collection**	**Where was the data collected? e*.g. home, clinic, workplace***	**Provider interviews were done in Kampala at a conference center being used for the randomization ceremony. Patient interviews were done at their local clinic in a private exam room.**
**15.**	**Presence of non-participants**	**Was anyone else present besides the participants and researchers?**	**A translator was present at the interviews.**
**16.**	**Description of sample**	**What are the important characteristics of the sample? *e.g. demographic data, date***	**The provider participants represented all 18 included health centers – a mixture of men and women of diverse ages. Patients were a mixture of HIV positive, early and late stages of TB treatment, and near even gender distribution men and women.**
**Data collection**			
**17.**	**Interview guide**	**Were questions, prompts, guides provided by the authors? Was it pilot tested?**	**A semi-structured interview guide was used for all interviews.**
**18.**	**Repeat interviews**	**Were repeat interviews carried out? If yes, how many?**	**No.**
**19.**	**Audio/visual recording**	**Did the research use audio or visual recording to collect the data?**	**The research was audio recorded.**
**20.**	**Field notes**	**Were field notes made during and/or after the interview or focus group?**	**Short-hand field notes were taken during the interviews and focus groups. These were completed immediately following the data collection.**
**21.**	**Duration**	**What was the duration of the interviews or focus group?**	**The focus groups ranged from 45-60 minutes and the interviews from 15 to 60 minutes.**
**22.**	**Data saturation**	**Was data saturation discussed?**	**Yes. After interviews were completed researchers gathered to determine if thematic saturation was achieved.**
**23.**	**Transcripts returned**	**Were transcripts returned to participants for comment and/or correction?**	**No.**
**Domain 3: analysis and findings**			
**Data analysis**			

**24.**	**Number of data coders**	**How many data coders coded the data?**	**2**
**25.**	**Description of the coding tree**	**Did authors provide a description of the coding tree?**	**No.**
**26.**	**Derivation of themes**	**Were themes identified in advance or derived from the data?**	**Themes were identified from the data in concordance with HCD methodology.**
**27.**	**Software**	**What software, if applicable, was used to manage the data?**	**No software was used other than word processers.**
**28.**	**Participant checking**	**Did participants provide feedback on the findings?**	**No.**
**Reporting**			
**29.**	**Quotations presented**	**Were participant quotations presented to illustrate the themes / findings? Was each quotation identified? e*.g. participant number***	**Yes - quotations were used to illustrate themes for the inspiration phase and quotes were identified.**
**30.**	**Data and findings consistent**	**Was there consistency between the data presented and the findings?**	**Yes – the insights and themes found during interviews and feedback sessions were used to refine future prototypes.**
**31.**	**Clarity of major themes**	**Were major themes clearly presented in the findings?**	**Yes – they are present in Table 1.**
**32.**	**Clarity of minor themes**	**Is there a description of diverse cases or discussion of minor themes?**	**As this paper focuses on the changes made because of the qualitative conclusions, minor themes are not discussed.**
