# Supplementary material for: Iterative Adaptation of a Tuberculosis Digital Medication Adherence Technology to Meet User Needs: Qualitative Study of Patients and Health Care Providers Using Human-Centered Design Methods
Source: JMIR Form Res. 2020 Dec 8;4(12):e19270. doi: 10.2196/19270 (PMC7755538; doi:10.2196/19270)
Supplement: Multimedia Appendix 2 [file formative_v4i12e19270_app2.docx]

**SUPPLEMENTARY MATERIALS
Supplementary Table 1: Focus-group Standardized Questionnaire**

Facilitator Name: ____________________________________________________

Participant Roles and Hospitals

****REMIND YOUR PARTICIPANTS:** Honest and critical feedback is encouraged. Please do not hesitate in telling us if you DISLIKE the prototype! It helps us immensely to design the best product for Uganda.**

Quotes should be labelled by who says them. For instance, if the TB Focal offers a suggestion, please mark that in your notes.

**Prototypes: Front Cover**

These prototypes have been designed to help provide an additional level of privacy and confidentiality. By being able to conceal their TB medications, as well as disguise it as something else, these prototypes are meant to subvert the stigma.

There are four front cover prototypes: 1) Imagery (textile and landscape); 2) “Daily Vitamins” label; 3) Calendar (monthly vs weekly view).

**IMAGERY (Textile and Landscape)**

| **Time** | **Activity/Questions** | **Notes** |
| --- | --- | --- |
| 3 mins | 1. Which one (textile or landscape) do you like better? Why? 2. What do you not like about the image(s)? 3. How might you change it? What images do you want to see? |  |

**“DAILY VITAMINS”**

| **Time** | **Activity/Questions** | **Notes** |
| --- | --- | --- |
| 3 mins | 1. What do you like about this prototype? 2. What do you not like about this prototype? 3. How might you change it? |  |

**CALENDAR**

| **Time** | **Activity/Questions** | **Notes** |
| --- | --- | --- |
| 3 mins | 1. What do you like about the calendar? 2. What do you not like about the calendar? 3. Which calendar format do you think makes more sense? 4. How might you change the calendar? |  |

**Prototypes: Inside Cover**

The inside cover of the pill pack is an opportunity for us to provide clear and concise instructions that can be understood by all patients (both text and images). This is also an opportunity to provide further education and information about TB or store important clinic/CHW/VHT information.

There are four inside cover prototypes: 1) Before and after imagery; 2) Meet your CHW or VHT; 3) TB Education customization; 4) TB education via cartoon and the instructions.

**BEFORE AND AFTER imagery**

| **Time** | **Activity/Questions** | **Notes** |
| --- | --- | --- |
| 5 mins | 1. What do you like about the image? 2. What do you not like about the image? 3. What message do you think the image sends? Is this the message we want to send to patients? (Is the image clear in the message it is trying to send?) 4. How might you change the image? |  |

**MEET YOUR VHT**

| **Time** | **Activity/Questions** | **Notes** |
| --- | --- | --- |
| 5 mins | 1. What do you like about this prototype? 2. What do you not like this prototype? 3. What information do patients want about their CHW/VHT? 4. How might you change this prototype? |  |

**TB EDUCATION CUSTOMIZATION**

| **Time** | **Activity/Questions** | **Notes** |
| --- | --- | --- |
| 5 mins | 1. What do you like about this prototype? 2. What do you not like this prototype? 3. What information do patients want to learn that we can add? 4. How might you change this prototype? |  |

**TB EDUCATION – CARTOON/COMIC**

| **Time** | **Activity/Questions** | **Notes** |
| --- | --- | --- |
| 5 mins | 1. What do you like about this prototype? 2. What do you not like this prototype? 3. How might you change this prototype? 4. Are there cartoons/comics that most Ugandan people would be able to relate to? |  |

**99DOTS INSTRUCTIONS**

| **Time** | **Activity/Questions** | **Notes** |
| --- | --- | --- |
| 5 mins | 1. Do the instructions clearly communicate what the patient needs to do? 2. Which icons communicate the information clearly? 3. How might you change or add to the instructions? 4. What are your thoughts on the colors of the icons? Particularly, background colors and color of human faces?   **TALLY HOW MANY PEOPLE LIKE EACH ICON:**  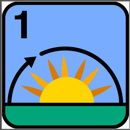 vs 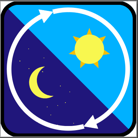   \|  \|  \| \| --- \| --- \|   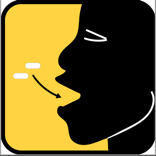 vs 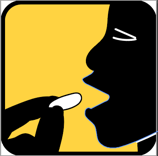   \|  \|  \| \| --- \| --- \|   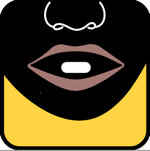 vs 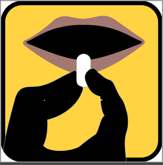   \|  \|  \| \| --- \| --- \|   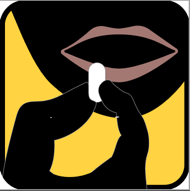 vs 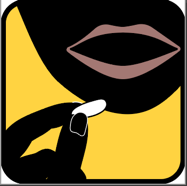   \|  \|  \| \| --- \| --- \|   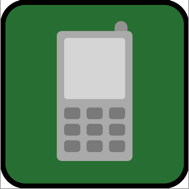vs 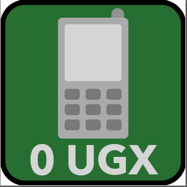   \|  \|  \| \| --- \| --- \|   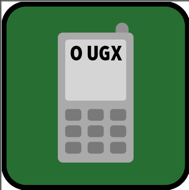 vs 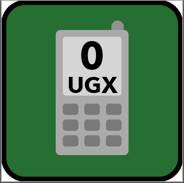   \|  \|  \| \| --- \| --- \| |  |

**Prototypes: Wayfinding**

These prototypes explore how to best guide patients to take their medications in a way that ensures that they take medicines in an order by which they call the toll free line everyday.

There are 4 types of prototypes: 1) Grid/draw your own; 2) Serpentine pattern; 3) Chevron pattern; 4) Complete the story

**GRID/DRAW YOUR OWN: Health workers box in the number of pills pts take each day.**

| **Time** | **Activity/Questions** | **Notes** |
| --- | --- | --- |
| 5 mins | 1. What do you like about this prototype? 2. What do you not like about this prototype? 3. How might you change this prototype? |  |

**SERPENTINE: arrows to guide pill-taking**

| **Time** | **Activity/Questions** | **Notes** |
| --- | --- | --- |
| 5 mins | 1. What do you like about this prototype? 2. What do you not like about this prototype? 3. How might you change this prototype? |  |

**CHEVRON – arrow-like design to guide the order of pill-taking**

| **Time** | **Activity/Questions** | **Notes** |
| --- | --- | --- |
| 5 mins | 1. What do you like about this prototype? 2. What do you not like about this prototype? 3. How might you change this prototype? |  |

**COMPLETE THE STORY: using a sentence to complete the “story” to guide the direction of pill-taking**

| **Time** | **Activity/Questions** | **Notes** |
| --- | --- | --- |
| 5 mins | 1. What do you like about this prototype? 2. What do you not like about this prototype? 3. How might you change this prototype? 4. What languages should the story be in? |  |

**Prototypes: Messaging**

**Devika will read aloud the different messages on projector. This will be a large group discussion.**

| **Time** | **Activity/Questions** | **Notes** |
| --- | --- | --- |
| 10 mins | 1. What messages would you want to add? 2. Which messages would you want to edit? 3. What languages should the messages be in? |  |
